# Neuronal activity inhibits mitochondrial transport only in synaptically connected segments of the axon

**DOI:** 10.3389/fncel.2024.1509283

**Published:** 2024-12-04

**Authors:** Tom Venneman, Pieter Vanden Berghe

**Affiliations:** Lab for Enteric NeuroScience (LENS), TARGID, KU Leuven, Leuven, Belgium

**Keywords:** axonal mitochondrial transport, neuronal activity, ratiometric calcium imaging, synaptic connections, transport regulation

## Abstract

Due to their large scale and uniquely branched architecture, neurons critically rely on active transport of mitochondria in order to match energy production and calcium buffering to local demand. Consequently, defective mitochondrial trafficking is implicated in various neurological and neurodegenerative diseases. A key signal regulating mitochondrial transport is intracellular calcium. Elevated Ca^2+^ levels have been demonstrated to inhibit mitochondrial transport in many cell types, including neurons. However, it is currently unclear to what extent calcium-signaling regulates axonal mitochondrial transport during realistic neuronal activity patterns. We created a robust pipeline to quantify with high spatial resolution, absolute Ca^2+^ concentrations. This allows us to monitor Ca^2+^ dynamics with pixel precision in the axon and other neuronal compartments. We found that axonal calcium levels scale with firing frequency in the range of 0.1–1 μM, whereas KCl-induced depolarization generated levels almost a magnitude higher. As expected, prolonged KCl-induced depolarization did inhibit axonal mitochondrial transport in primary hippocampal neurons. However, physiologically relevant neuronal activity patterns only inhibited mitochondrial transport in axonal segments which made connections to a target neuron. In “non-connecting” axonal segments, we were unable to trigger this inhibitory mechanism using realistic firing patterns. Thus, we confirm that neuronal activity can indeed regulate axonal mitochondrial transport, and reveal a spatial pattern to this regulation which went previously undetected. Together, these findings indicate a potent, but localized role for activity-related calcium fluctuations in the regulation of axonal mitochondrial transport.

## Introduction

Though the brain only makes up 2% of our body by weight, it consumes 20% of our total resting energy production (Mink et al., 1981). This energy is mainly produced by mitochondria in the form of ATP through oxidative phosphorylation (Kann and Kovács, 2007). Remarkably, a single cortical neuron is estimated to consume around 4.7 billion ATP molecules per second (Zhu et al., 2012). Most of this energy is spent on reversing the ion influxes that underlie synaptic signaling and action potential (AP) firing (Attwell and Laughlin, 2001). Additionally, mitochondria play a key role in calcium homeostasis and signaling, by sequestering Ca^2+^ influx and buffering [Ca^2+^]_i_ fluctuations (Kann and Kovács, 2007; Vaccaro et al., 2017). Due to their large scale and uniquely branched architecture, neurons critically rely on active transport of mitochondria in order to match energy production and calcium buffering to local demand (Chen and Chan, 2006). Consequently, defective mitochondrial trafficking is implicated in various neurological (d’Ydewalle et al., 2011; Alexander et al., 2000; Civera-Tregón et al., 2021) and neurodegenerative diseases (Sheng and Cai, 2012; Burté et al., 2015).

Long-range mitochondrial transport occurs along microtubule tracks. In the axonal compartment, these cytoskeletal polymers are uniformly oriented plus-end out. Therefore, kinesin motors exclusively power anterograde axonal transport, while dynein motors propel cargo in the retrograde direction (Kevenaar and Hoogenraad, 2015). Hence, time-lapse imaging of mitochondrial transport in single axons is a powerful method to study transport regulation, enabling direct quantification of effects on either family of motors (van Steenbergen et al., 2022). These molecular motors are supported by various adaptors, facilitating regulation (Sheng, 2017). The result is a highly complex and dynamic system, characterized by “saltatory” movement; mitochondria frequently pause, change velocity and switch directions (Vanden Berghe et al., 2004).

A key signal regulating mitochondrial transport is intracellular calcium. Elevated Ca^2+^ levels have been demonstrated to inhibit mitochondrial movement in many cell types, including neurons (Rintoul et al., 2003; Yi et al., 2004; Saotome et al., 2008; Wang and Schwarz, 2009). Two molecular mechanisms have been identified so far; one involves an adaptor complex, called the MIRO1-TRAK2-kinesin complex (Saotome et al., 2008; Wang and Schwarz, 2009; MacAskill et al., 2009), another involves a static anchor protein, syntaphilin (Kang et al., 2008; Chen and Sheng, 2013). According to popular theory, these mechanisms recruit mitochondria to sites with increased Ca^2+^ buffering and energy demands, by inhibiting movement where Ca^2+^ levels are elevated (Sheng, 2017). However, these inhibitory mechanisms were predominantly demonstrated using sustained [Ca^2+^]_i_ elevations, e.g., following a 3–10 min exposure to KCl, calcimycin or glutamate (Rintoul et al., 2003; Yi et al., 2004; Saotome et al., 2008; Wang and Schwarz, 2009; MacAskill et al., 2009; Kang et al., 2008; Chen and Sheng, 2013). Indeed, Ca^2+^ levels can spike a 100-fold during neuronal activity; however, they are rarely sustained under physiological conditions (Chen et al., 2013). Hence, it is currently unclear to what extent calcium-signaling regulates axonal mitochondrial transport during realistic neuronal activity patterns.

In this study, we confirm that neuronal activity can indeed regulate axonal mitochondrial transport, but we reveal a spatial pattern to this regulation which went previously undetected. Prolonged KCl-induced depolarization of the neuron did inhibit axonal mitochondrial transport in line with previous reports. However, realistic neuronal activity patterns only inhibited mitochondrial transport in axonal segments connected to a target neuron, not in segments without these connections. Together, the findings presented in this paper indicate a potent, but localized role for activity-related calcium signaling in the regulation of axonal mitochondrial transport.

## Results

### Activity-related calcium spikes are not sufficient to halt axonal mitochondrial transport

**Figure 1:**
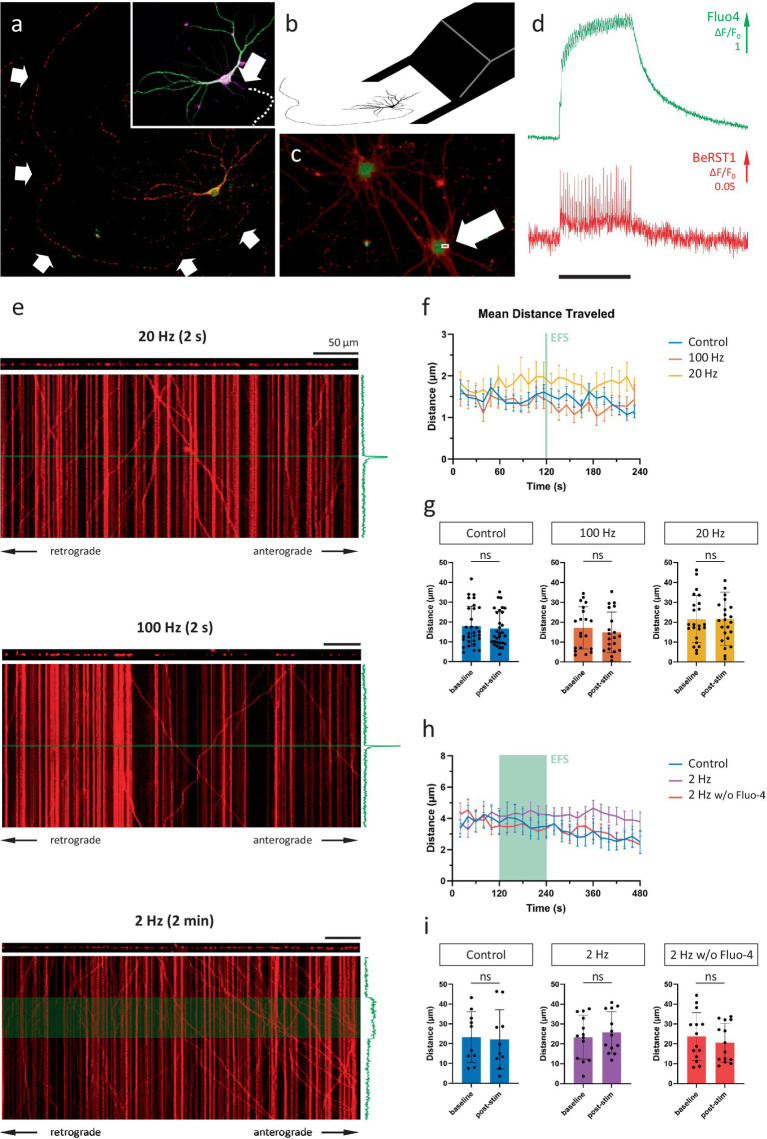
Axonal mitochondrial transport is unaffected by neuronal activity patterns. **(A)** Representative field of view (FOV) for near-simultaneous recording of calcium activity (Fluo-4) and axonal mitochondrial transport (mitoTracker Red). Arrows mark axonal fiber. Inset: MAP2 (green), AnkG (magenta) and Tau immunolabeling to verify axonal identity and origin. Arrow indicates axon initial segment. Dotted line indicates axonal path. **(B)** Schematic representation of bipolar electrode positioning. **(C)** Primary hippocampal neurons loaded with cytosolic calcium indicator Fluo-4 (green) and membrane-bound voltage sensor BeRST1 (red). Arrow indicates 4 × 2 pixel ROI used for high-frequency scanning. **(D)** Calcium and voltage responses during 20 Hz stimulation (black bar marks stimulation period = 2 s). **(E)** Kymographs of representative recordings of the 3 main stimulation paradigms; 20 Hz 2 s, 100 Hz 2 s and 2 Hz 2 min. The axonal segment is shown above each kymograph and the axonal Fluo-4 signal along the time axis. Green line/box indicates stimulation period. Scale bars = 50 μm. **(F)** Binned distance over time reveals no effect on transport during/following activity. **(G)** Quantification of distance traveled during baseline period (1st half of recording) vs. post-stimulation (2nd half of recording). Mann–Whitney *U* test, *p* = 0.62 (ctr), 0.49 (100 Hz), 0.96 (20 Hz), *n* = 30 recordings & 255 mobile mitochondria (ctr), 20 recordings & 145 mobile mitochondria (100 Hz), 23 recordings & 205 mobile mitochondria (20 Hz). **(H,I)** Idem panel **F,G**. For these recordings, the first 2 min (=baseline) were compared to the 2 min stimulation period, with and without Fluo-4 present. Mann–Whitney *U* test, *p* = 0.74 (ctr), 0.48 (2 Hz), 0.60 (2 Hz w/o Fluo-4), *n* = 10 recordings, 141 mobile mitochondria (ctr), 13 recordings, 196 mobile mitochondria (2 Hz), 14 recordings, 189 mobile mitochondria (2 Hz w/o Fluo-4).

To investigate to what extent Ca^2+^ fluctuations impact axonal mitochondrial transport under physiologically relevant conditions, we optimized a set-up to simultaneously record mitochondrial transport and calcium dynamics in single axons of primary hippocampal neurons in sparse cultures (Figure 1A). Axon origin and identity were verified via *post hoc* immunolabeling (Figure 1B, inset), enabling the distinction between anterograde and retrograde transport. To induce the firing patterns of choice, precise electrical field stimulation (EFS) was performed by carefully positioning a custom-made bipolar electrode around the cell soma (Figure 1B). Time-lapse imaging at high temporal resolution (~300 Hz), using the voltage sensor BeRST1 (Huang et al., 2015) in combination with the calcium indicator Fluo-4, confirmed the tight coupling between action potential firing and calcium spikes (Figures 1C,D).

**Figure 2:**
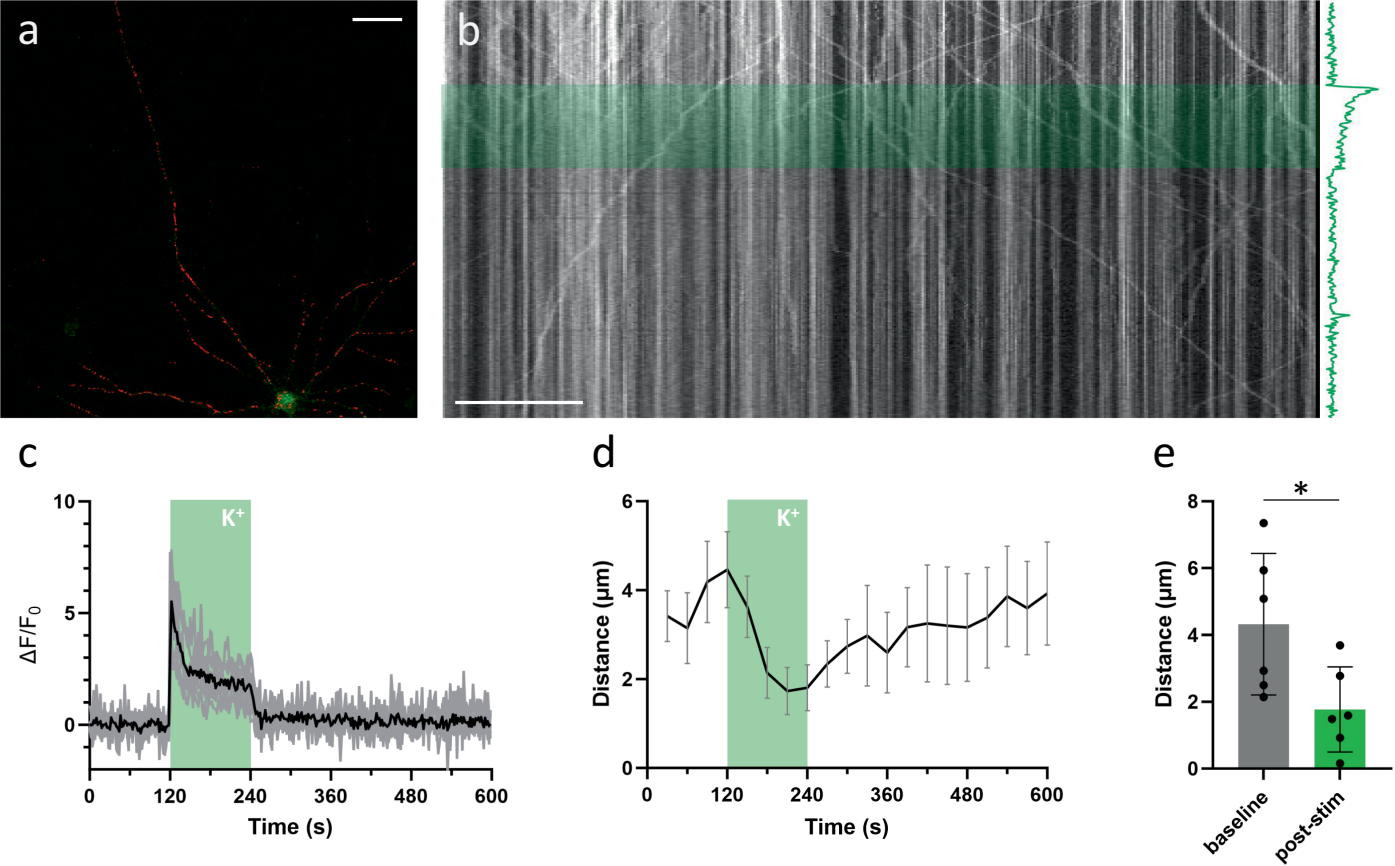
KCl perfusion inhibits axonal mitochondrial transport. **(A)** Example neuron, loaded with mitoTR and Fluo-4 (Scale bar = 50 μm). **(B)** Kymograph from axonal segment. The KCl perfusion period (2 min) is marked in green. The axonal Ca^2+^ response is shown along the time-axis. **(C)** Mean axonal Ca^2+^ response to KCL perfusion in black, individual traces in gray. **(D)** Axonal transport over time (quantified as the mean distance traveled per mitochondrion, binned every 30 s. Error bars = SEM). KCl perfusion period (2 min) is marked in green. (*n* = 6 recordings, 8 segments, 97 mobile mitochondria). **(E)** Statistical comparison of mean distance traveled between baseline period (minute before stimulation) vs. last minute of stimulation period. Mann–Whitney *U* test (*p* = 0.04). Error bars = STD.

Since KCl-induced depolarization has been used previously to demonstrate the calcium-dependent inhibition of mitochondrial transport, we first repeated this finding (Figures 2A–C). In line with previous reports, a 2-min KCl perfusion resulted in a substantial, yet temporary, decrease in axonal mitochondrial transport (Figures 2D,E). Such a sustained depolarization, however, does not perfectly represent the calcium spikes which occur during neuronal activity. Therefore, to more closely match physiological conditions, we then tested how activity patterns of different frequencies affected transport. Analysis of transport parameters was performed based on manual tracings of mitochondrial trajectories in kymographs, 2D projections of the axonal segment (Figure 1E). Surprisingly, none of these movement parameters were affected by the activity patterns that were tested, even at the highest stimulation frequency (100 Hz). The quantification of the unidirectional parameter “distance traveled over time” is shown in Figures 1F,G, demonstrating the lack of any effect. As the methodology also allows analyzing anterograde and retrograde directions of transport separately, we also compared direction specific parameters, but neither of these were affected (Supplementary Figure S1A). Other movement parameters, such as mobile fraction (Supplementary Figure S1B) and motor velocity (Supplementary Figure S1C) were also unaffected.

To assess whether the inhibitory effect scales with the duration of the activity pattern, rather than the frequency, we tested another stimulation paradigm during which cells were induced to fire for an extended period of time (2 min, 2 Hz). Again, there was no effect on the distance traveled by mitochondria (Figures 1H,I), or any of the other transport parameters (not shown). To rule out the potential chelation of calcium by the indicator itself, the experiment was repeated in the absence of a calcium indicator. Likewise, axonal mitochondrial transport, in either direction, remained unaffected by the induced activity patterns (Figures 1H,I).

### Axonal calcium levels scale with firing frequency in the range of 0.1–1 μM

**Figure 3:**
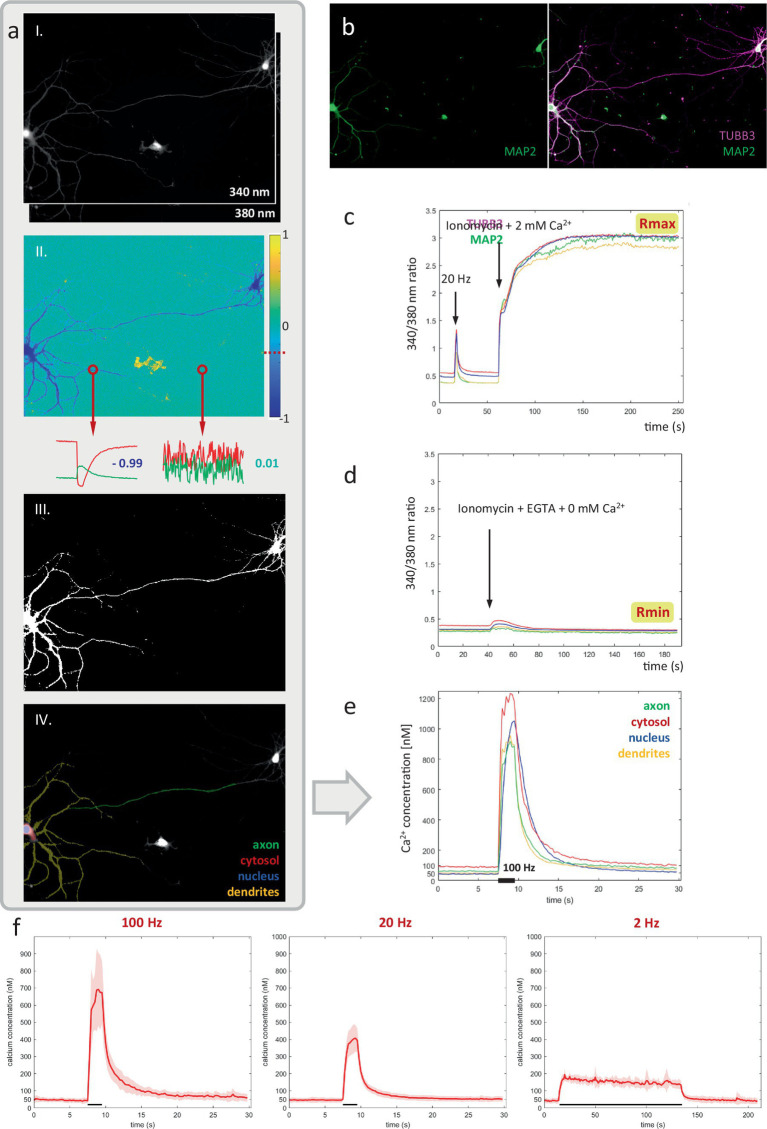
A robust pipeline for measuring absolute calcium concentrations in the axon and other neuronal compartments. **(A)** Key steps in the pipeline. From top to bottom: **(i)** near-simultaneous widefield imaging at 340 and 380 nm, **(ii)** inverse correlation-based thresholding, **(iii)** resulting neuronal mask, **(iv)** used-defined compartment-ROIs. (B) Immunolabeling for MAP2, TUBB3 and DAPI to distinguish compartments: axon, dendrites, soma and nucleus. **(C,D)** Calibration of 340/380 nm ratio to absolute calcium concentration in living neurons, using ionomycin to saturate and deplete intracellular calcium. **(E)** Absolute calcium responses measured in each neuronal compartment for representative recording of a primary hippocampal neuron shown in panel **(A)**, stimulated at 100 Hz for 2 s. **(F)** average axonal responses to 3 main stimulation paradigms. *N* = 6 cells (100 Hz), 13 cells (20 Hz), 6 cells (2 Hz).

In contrast to KCl-induced depolarization (Figure 2), activity-induced calcium elevations did not inhibit axonal mitochondrial transport (Figure 1). A potential explanation might be that the calcium spikes which occur during spontaneous firing (and EFS-induced activity patterns) are insufficiently high to trigger this inhibitory effect in the axon. To this end, we created a robust method to quantify absolute Ca^2+^ concentrations in neuronal compartments with high spatial precision, using the ratiometric Ca^2+^ indicator Fura-2 (Figure 3A).

**Figure 4:**
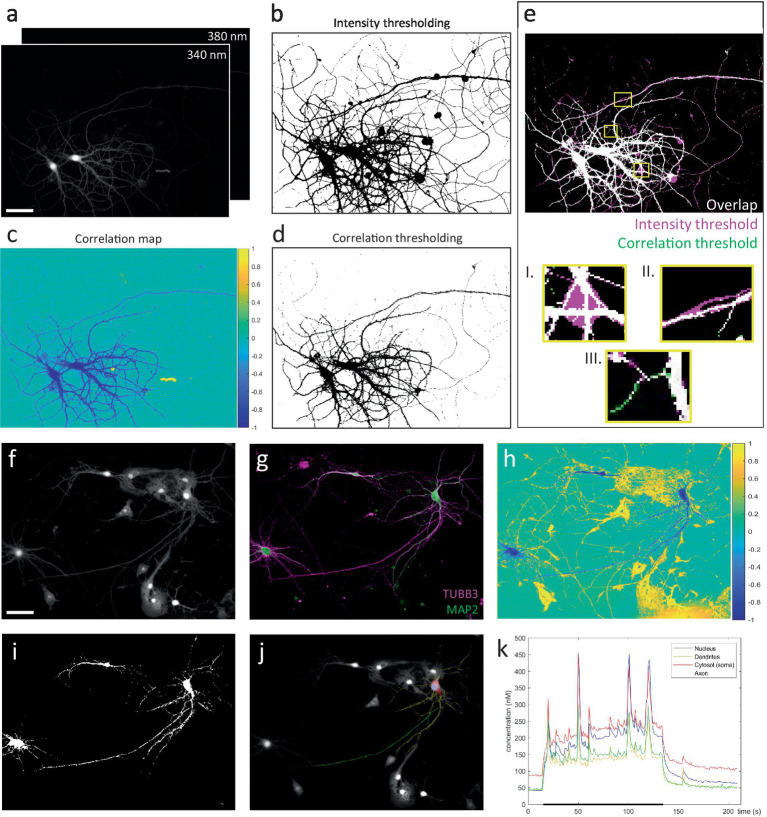
Correlation-based thresholding vs. intensity-based approach for the segmentation of ratiometric image stacks. **(A)** Raw image stacks (scale bar = 50 μm). **(B)** Segmentation mask obtained by conventional manual thresholding, performed on a median projection of each image stack. The resulting masks were then combined to generate the final mask. **(C)** Correlation map, generated by pixel-wise correlation of 340 & 380 nm pixel traces. **(D)** Segmentation mask based on correlation thresholding. For all recordings included in the dataset for Figure 3, a correlation threshold of −0.3 reliably segmented the neuronal silhouette. **(E)** Comparison of intensity (magenta) vs. correlation-based (green) segmentation methods (white = overlap). Correlation-based thresholding easily excludes pixels where no reliable measurement can be made and which would otherwise contaminate quantification, e.g., fluorescent debris **(I.)** and non-responsive fibers **(II.)**. Moreover, responsive yet low-intensity fibers are more easily segmented by correlation-based thresholding owing to their negative correlation values **(III.)**. **(F)** Example recording that represents near-impossible task for intensity-based segmentation of a neuron surrounded by various other cells (scale bar = 50 μm). **(G)** Immunocytochemistry to distinguish neuronal compartments. **(H)** Correlation map. Neuron of interest can be identified based on its negative correlation values. Surrounding cells (and debris) earn (neutral or) positive correlation between the 340 & 380 nm pixel traces, due to cell movement and signal bleaching. **(I)** Segmentation mask (threshold = −0.3). **(J)** User-defined compartmental ROIs. **(K)** Compartment-specific Ca^2+^ responses during a 2-min train of action potentials at 2 Hz (black bar).

First, image stacks recorded at 340 nm & 380 nm excitation were registered, and background-corrected by subtracting identically acquired images in an identical sample preparation containing imaging medium but no cells. Next, we exploit the tight inverse correlation between the 340 & 380 nm signals exhibited by pixels within the responding cell, to segment a mask of the neuron (Figure 3A). Compared to intensity-based thresholding, this method enables near-perfect separation from non-responsive cells or fibers, moving cells and debris (Figure 4). The resulting binary mask is used to define compartment-specific ROIs, guided by post-hoc immunolabeling using anti-MAP2 and anti-TUBB3 antibodies (Figure 3B). Since ratiometric methods are sensitive to background contributions, we also corrected the extracted ROI-signals for their compartment-specific autofluorescence contribution, which was measured in unloaded neurons (not shown).

Finally, absolute calcium concentrations were calibrated based on a set of reference measurements, performed in primary hippocampal neurons under identical conditions. Briefly, the maximal and minimal 340/380 nm ratios (*R*_max_ & *R*_min_) were determined by intracellular measurement in living neurons, using ionomycin and varying concentrations of external Ca^2+^ to saturate and deplete intracellular calcium levels, respectively (Figures 3C,D).

To account for differences in excitation (340 & 380 nm) and efficiency of the Ca^2+^-bound and Ca^2+^-free Fura2-signals, a ROI or compartment-specific correction value alpha (*α*) is used to calculate the effective dissociation constant *K*_eff_:

$$K_{\mathrm{eff}}=K_{D}*\frac{\left(R_{\text{max}}+\alpha \right)}{\left(R_{\text{min}}+\alpha \right)}$$

which is used to calculate the actual intracellular Ca^2+^ concentration, using:

$$\left[{Ca}^{2+}\right]=K_{\mathrm{eff}}*\frac{\left(R-R_{\text{min}}\right)}{\left(R_{\text{max}}-R\right)}$$

**Figure 5:**
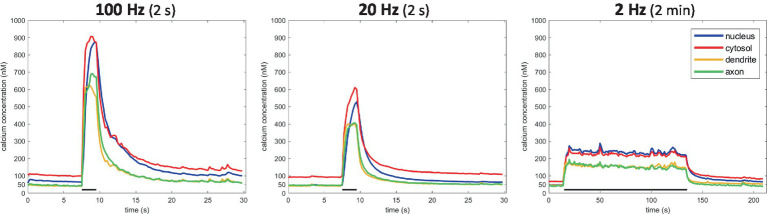
Compartment-specific Ca^2+^ responses to 3 stimulation types. *N* = 6 cells (100 Hz), 13 cells (20 Hz), 6 cells (2 Hz).

This approach enabled us to monitor Ca^2+^ dynamics in great detail, within different compartments of the same neuron (Figure 3E). A ~ 50 nM baseline was observed in all compartments, except for the cytosolic one, where values ranged between 50–100 nM (Figure 5). Calcium responses differed as well between compartments. Ca^2+^ responses typically rose more slowly in the nucleus than in the cytosol, where the concentration reached higher levels than anywhere else in the neuron. A high degree of variability in response amplitude was observed within the dendritic tree, compared to low variability in the axonal response. Figure 3F shows the average absolute calcium responses in the axonal compartment to the 3 types of activity patterns used in previous transport experiments: during a 100 Hz 2 s burst, Ca^2+^ reached 713 ± 211 nM, for 20 Hz 2 s burst, 419 ± 76 nM and for a 2 Hz 2 min period, 182 ± 17 nM.

### Activity regulates mitochondrial transport in axonal segments that connect to target neurons

Previous reports have estimated the IC_50_ value of calcium’s inhibitory effect on mitochondrial transport to be in the range of 400 nM (Yi et al., 2004; Saotome et al., 2008; MacAskill et al., 2009). The firing patterns used in our experiments induced axonal calcium levels exceeding this threshold (Figure 3). Hence, if activity-dependent inhibition of transport was solely dependent on axonal calcium levels, we should have observed a cessation (or substantial decrease) in mitochondrial transport. However, in contrast to KCl-induced depolarization, realistic firing patterns did not inhibit axonal mitochondrial transport (Figure 1), despite inducing sufficiently high Ca^2+^ levels.

**Figure 6:**
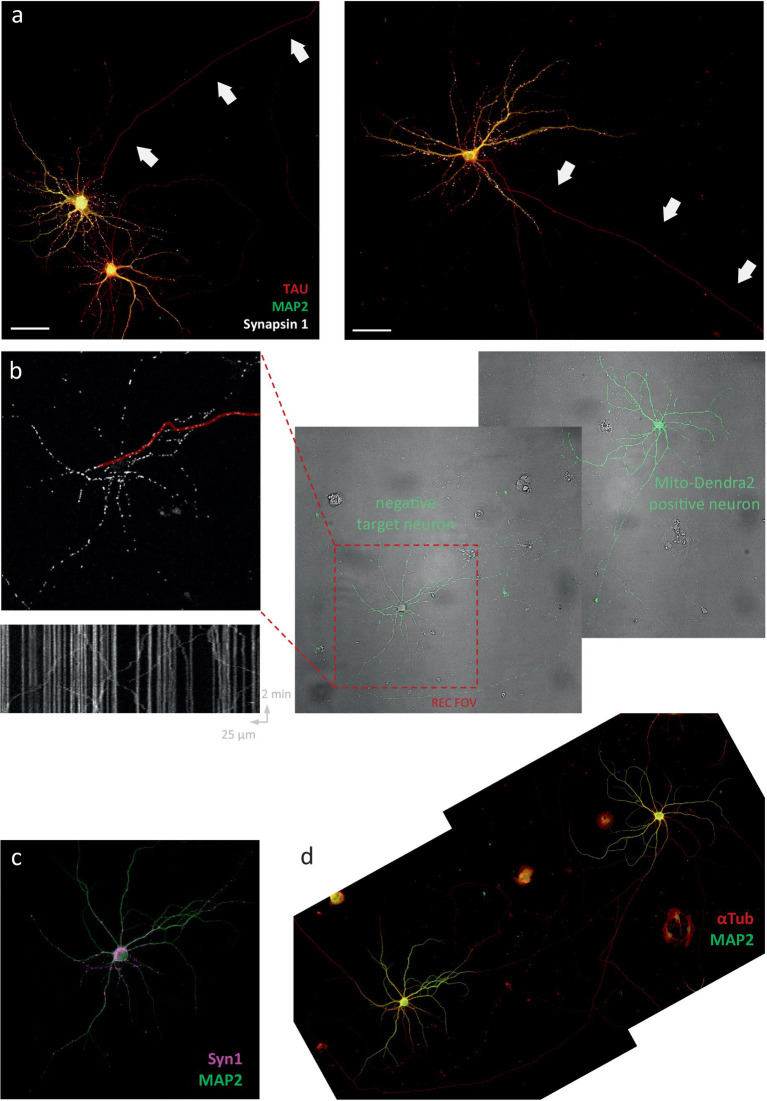

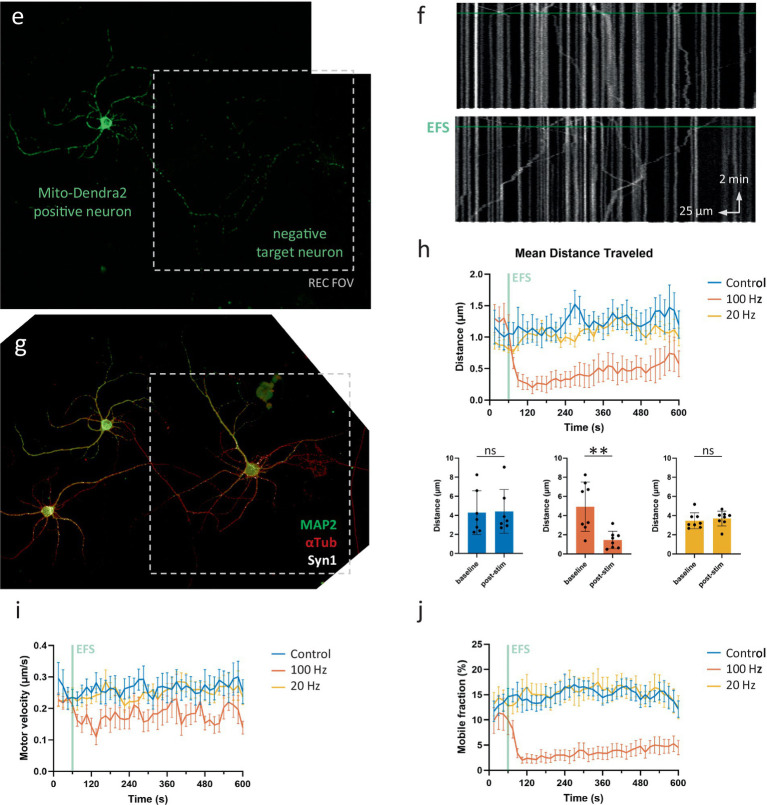
Activity regulates mitochondrial transport in axonal segments that connect to target neurons. **(A)** Representative FOVs in sparse neuronal cultures. Arrows indicate lack of pre-synaptic sites in non-connecting axonal segments traditionally used for detailed transport measurements. Scale bars = 50 μm. **(B)** Overview protocol to measure axonal mitochondrial transport in connecting axonal segments (control recording, no stimulation). Right to left: mixed cultures of wildtype and mito-dendra2-positive neurons. FOV is chosen at target neuron (red square). Axonal segments are traced (example marked in red) to produce kymographs for analysis. **(C,D)** Immunolabeling for MAP2 (green), αTub (red) and Syn-1 (magenta) is used to confirm the connection. **(E–G)** Idem for a representative neuron stimulated at 100 Hz for 2 s. **(F)** Kymograph shows mitochondria halting shortly after electrical field stimulation (EFS). **(H)** Quantification of mean distance traveled over time and statistical comparison between baseline period vs. 1 min after stimulation. Mann–Whitney *U* test, *p* = 0.80 (ctr), 0.0047 (100 Hz) and 0.28 (20 Hz). *N* = 7 recordings, 21 segments, 360 mobile mitochondria (ctr), 8 recordings, 23 segments, 212 mobile mitochondria (100 Hz), 8 recordings, 30 segments, 469 mobile mitochondria (20 Hz). **(I,J)** Quantification of velocity and mobile percentage over time.

Since one of the proposed functions of this regulatory mechanism is to recruit mitochondria to pre (Vaccaro et al., 2017; Chen and Sheng, 2013; Obashi and Okabe, 2013) -and post (MacAskill et al., 2009; Li et al., 2004)-synaptic locations, we hypothesized that its action might be locally constrained within the axon. Therefore, we set out to test whether neuronal activity affects mitochondrial transport in more distally located segments, where it branches and makes connections to another neuron. To this end, we adjusted our experimental approach. Instead of quantifying transport in non-connecting (Figure 6A) segments of the axon (which lack functional synapses), transport recordings were performed in the connecting segments of mito-dendra2-positive neurons in mixed cultures. These mixed cultures were identical in cell density, but made from a 50:50 mix of wildtype to mito-dendra2-positive neurons (Figure 6B). This allowed us to quantify mitochondrial transport specifically in positive axonal segments connecting to a negative target cell. Post-hoc immunolabeling was used to confirm the physical interaction between pre-and post-synaptic cells (Figures 6C,D).

In these connecting axonal segments, high frequency activity patterns (100 Hz, 2 s) resulted in a clear inhibition of mitochondrial transport (Figures 6E–H). This decrease was mainly achieved through halting mitochondria rather than slowing down their movements, as evidenced by a peak in pause events and a decrease in mobile fraction (Figure 6J), compared to the smaller effect on motor velocity (Figure 6I). Despite the relatively large inhibitory effect on some movement parameters, transport levels quickly recovered even during the length of the recording. Indeed, “mean distance traveled” and “mobile fraction” recovered to 54.2 and 45.4% of their baseline values, respectively, by the end of the recording, indicating the inhibitory effects are temporary in nature. No effect on transport was observed following a 20 Hz (2 s) burst of activity (Figure 6H).

Interestingly, the connecting segments imaged in this experiment were often located as close to the soma as their non-connecting counterparts in the first set of experiments. Thus, the lack of inhibitory action in this first set of experiments cannot be attributed to their more “proximal” distance. In other words, the connecting neuron was often located in proximity to the mito-dendra2-positive neuron (as in the example shown in Figures 6E–G), meaning that the inhibition as a result of neuronal activity is associated with the presence of a connection, not its distance from the soma.

## Discussion

Calcium signaling serves a potent role in the regulation of mitochondrial transport and distribution (Sheng, 2017). Since intracellular calcium levels fluctuate during neuronal activity, we set out to study how action potential firing influences axonal mitochondrial transport. Surprisingly, we found no evidence that mitochondrial transport is affected by activity-related calcium fluctuations in non-connecting axonal segments (Figure 1). In axonal segments that do connect to target neurons however, neuronal activity was able to inhibit mitochondrial transport (Figure 6). This spatial pattern might suggest that *in vivo*, neuronal activity similarly does not affect mitochondrial transport along the majority of the projecting axon’s length, but does so only locally, where the axon makes its connection to target cells.

There are likely multiple reasons as to why this pattern was not detected in previous studies. The first is related to culture density. EFS has been used before to demonstrate activity-dependent inhibition of axonal mitochondrial transport (Obashi and Okabe, 2013). However, we elected to perform our first set of experiments in single axons in highly sparse cultures, where axons can extend for millimeters before connecting to a target neuron. This approach offers key advantages in terms of accessibility for imaging and sample manipulation, as well as analysis strategies. As a consequence, transport recordings biased towards non-connecting axonal segments. As a result, activity-related calcium elevations initially appeared insufficient to halt axonal mitochondrial transport, and finally led us to test a region-specific hypothesis.

**Figure 7:**
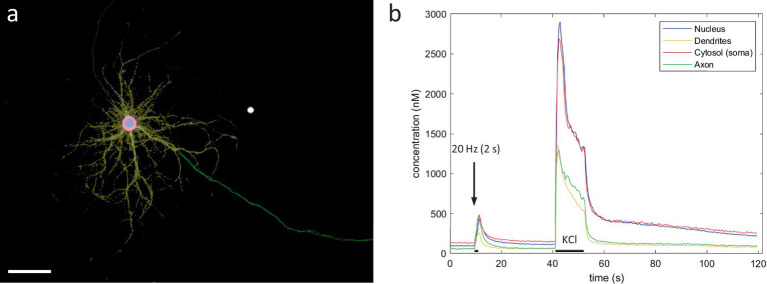
Compartment-specific Ca^2+^ responses to KCl perfusion. **(A)** User-defined compartmental ROIs (scale bar = 50 μm). **(B)** Compartment-specific Ca^2+^ responses after electrical field stimulation (20 Hz 2 s) and KCl perfusion (11.5 s).

Secondly, calcium’s inhibitory effect on mitochondrial transport in neurons has been demonstrated using stimuli of very different proportions. Most often, calcium-dependent inhibition of mitochondrial transport is demonstrated using stimuli, that produce elevations in calcium which surpass those observed during neuronal activity, in both concentration and duration (Rintoul et al., 2003; Yi et al., 2004; Saotome et al., 2008; Wang and Schwarz, 2009; MacAskill et al., 2009; Chen and Sheng, 2013). Similarly, when we applied KCl-depolarization, we observed a decrease in axonal mitochondrial transport, independent of the presence of a nearby target neuron (Figure 2). However, the axonal calcium levels induced by KCl-depolarization are almost a magnitude higher than those observed during realistic firing patterns (Figure 7).

This might suggest the existence of an alternative pathway, which also results in the inhibition of transport, but is triggered by a much higher calcium load. It is possible that such a mechanism could serve a neuroprotective role when calcium levels exceed their physiological range (Chang et al., 2006). Indeed, calcium-sensitive adaptors to the motor-complex have been demonstrated to protect against excitotoxicity associated with excessive calcium influx (Wang and Schwarz, 2009). Alternatively, the cessation of movement under such pathological conditions might result from an indirect effect on mitochondrial function, blocking ATP production. Similarly, calcium overload during epileptic seizures can lead to excessive Ca^2+^ uptake by mitochondria, attenuating their membrane potential, the main driver of ATP production (Kann and Kovács, 2007). Hence, energetic failure could have contributed to the inhibition of ATP-driven transport following some experimental manipulations. In light of these findings it is interesting to note that it took ca. 2 min to reach the highest level of inhibition following KCl-induced depolarization, but only ca. 30 s following the EFS-induced activity patterns. Together, these findings indicate calcium’s role as a regulatory cue is more complex than previously thought, potentially serving a different purpose during realistic firing, than when homeostasis is threatened.

In an effort to study calcium dynamics in the axon, we created a robust method to quantify absolute calcium concentrations with high spatial precision using the ratiometric Ca^2+^-indicator Fura-2. This method was then used to describe the calcium responses in each neuronal compartment following the EFS-induced activity patterns employed during our transport recordings (Figure 5). Axonal calcium levels were observed to scale with firing frequency in the range of 0.1–1 μM (Figure 3F). Ratiometric imaging, moreover, did not reveal notable differences in the calcium response amplitude along the length of the axon (i.e., low variability in response amplitude). Hence, the spatial pattern identified in this work, is unlikely to arise due to differences in calcium response amplitude between axonal segments. One potential explanation might be a correlative distribution of syntaphilin (Chen and Sheng, 2013), an axon-specific mitochondrial docking protein capable of halting transport in Ca^2+^-dependent manner (i.e., low expression in non-connecting segments).

**Figure 8:**
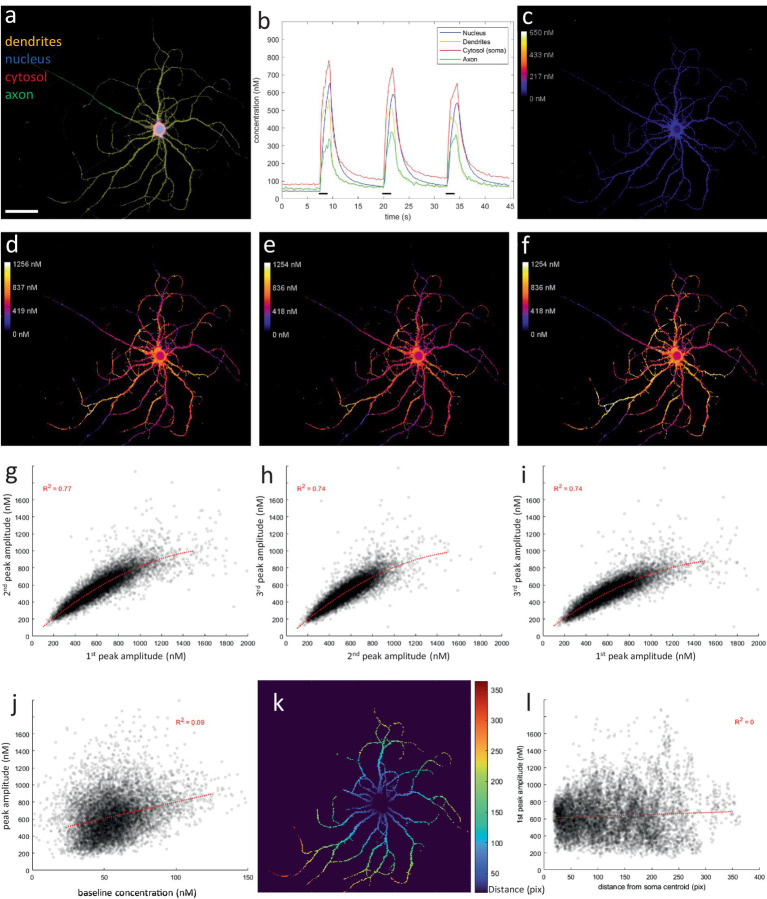
Ratiometric Ca^2+^ imaging reveals complex spatial patterns within dendritic compartment. **(A)** User-defined compartment ROIs (scale bar = 50 μm). **(B)** Compartment-specific Ca^2+^ responses to repeated 20 Hz 2 s stimulation (black bars, 3×). **(C)** Baseline map (average of first 30 frames or 7.5 s). A baseline of ca. 50 nm is observed in all compartments, except the cytosol, which measured ca. 100 nM. **(D–F)** Peak amplitude maps for each repetition of the stimulation (average of 8 frames during stimulation). Upon stimulation, Ca^2+^ response amplitude is highly variable throughout the dendritic tree. However, this complex spatial pattern is reproduced upon repeated stimulation. **(G–I)** Scatterplots demonstrating the correlation between response amplitudes during repeated stimulations. **(J)** Local response amplitudes are not correlated to baseline levels. **(K)** Distance map, indicating the quasi-Euclidean distance (in pixels) from each ROI pixel to the center of the soma, as the shortest distance traversed within the constraints of the dendritic mask. **(L)** Local response amplitudes are not correlated to the distance from the soma. Idem for baseline map (data not shown).

The main focus of this study was on the axonal compartment. However, ratiometric imaging also provided insights into the calcium dynamics in other neuronal compartments (Figure 5). At rest, a baseline of ca. 50 nM was maintained in all compartments (Figures 8A–C), in accordance with previous reports (Schiller and Sakmann, 1995; Maravall et al., 2000; Ali and Kwan, 2019). Except in the cytosol, were a higher and more variable baseline was measured of ca. 50–100 nM. “Peak amplitude maps,” produced by an analogue pipeline based on identical calculations performed per pixel instead of per ROI-average signal, also revealed a high variability in the calcium responses between dendritic branch segments (Figure 8D). Moreover, this complex pattern was reproduced during repeated stimulations (Figures 8E–I). Local response amplitude, at any given dendritic location, was not correlated to baseline amplitude, nor was it determined by its distance from the soma (Figures 8J–L). These patterns explain the higher variability in dendritic calcium responses observed via the ROI-based approach. Furthermore, the dendritic calcium levels evoked by the activity patterns tested in this study were likely sufficient to trigger transport inhibition, based on previous estimates of its IC_50_ value (MacAskill et al., 2009). Since branches with high response amplitudes are more likely to halt, and thus accumulate, mitochondria during neuronal activity, these dendritic response patterns might be an important cue to regulate the mitochondrial distribution in the dendritic compartment.

## Methods

### Primary hippocampal cultures

1–2 day old C57BL/6J mouse pups were quickly decapitated before dissection. Hippocampi were dissected in Sylgard dishes containing cold sterile Hank’s buffered salt solution (HBSS in mM: 5.33 KCl, 0.44 KH_2_PO_4_, 137.93 NaCl, 0.34Na_2_HPO_4_0.7H_2_O, 5.56 D-glucose and 10 HEPES). The tissue was incubated in 0.25% trypsin-ethylenediaminetetraacetic acid (EDTA) (Gibco) supplemented with 200 U/mL DNase (Roche) for 10 min at 37°C. After three consecutive wash steps with washing buffer [neurobasal medium (Invitrogen) supplemented with 1.59 mg/mL BSA, 0.5% penicillin/streptomycin, 5 mg/mL glucose, 5.5 mg/mL sodium pyruvate, and 200 U/mL DNAaseI] the tissue was mechanically dissociated by trituration. After centrifugation, cells were resuspended and plated on 18 mm diameter coverslips, coated with poly-D-Lysine, suspended over a glial support layer using paraffin dots. The glial support layer was derived using an identical protocol on midbrain and cortical tissue harvest from the same pup. Primary hippocampal neuron-glia co-cultures were grown in a 37°C, 5% CO_2_ incubator in Neurobasal-A media (Thermo Fisher Scientific) supplemented with 0.5% penicillin/streptomycin (Lonza), 2% B27 (Gibco), 0.02 mg/mL insulin (Sigma), 50 ng/mL nerve growth factor (Alomone Labs), and 0.5 mM Glutamax (Thermo Fisher Scientific). Half of the medium volume was replaced every 3 days. Imaging was performed after 7–10 days *in vitro*. We generated an in-house colony neuron specific mito-dendra2 transgenic mice, by crossing Thy1-Cre mice with ^fl/fl^mito-dendra2 reporter mice. These mice were used to source the mito-dendra2-positive primary hippocampal neurons. All procedures were approved by the Animal Ethics Committee of the University of Leuven (Belgium). Ethical Committee number = P110/2020.

### Immunocytochemistry

Coverslips were fixed in 4% PFA for 20 min, permeabilized and blocked in 0.5% triton-x solution with host (donkey) serum, labeled with primary antibodies at 4°C overnight (see table below), and finally incubated with secondary antibodies (1:1,000) at room temperature for 2 h.

**Table 1:** 

Primary antibody	Dilution	Purpose	Reference number
ch-MAP2	1:2,500–5,000	To define dendritic compartment	ab5392 (Abcam)
g-TAU C17	1:500	To distinguish axonal compartment in combination with MAP2	Sc-1995 (Santa Cruz)
m-ANK G	1:250	To define axon initial segment	Clone N106/20 (Antibodies Inc.)
rb-TUBB3	1:1,000	To distinguish axonal compartment in combination with MAP2	ab52623 (Abcam)
r-alphaTub	1:500	To distinguish axonal compartment in combination with MAP2	MA1-80189 (Thermo Fisher)
r-Syn-1	1:500	Pre-synaptic marker	ab64581 (Abcam)

### Axonal mitochondrial transport imaging

Time-lapse imaging of axonal mitochondrial transport and calcium dynamics was performed using a 25× objective lens on a Zeiss LSM 780 confocal microscope, at 37°C in HEPES buffered solution containing in mM; 140 NaCl, 5 KCl, 10 HEPES, 2 CaCl_2_, 2 MgCl_2_ and 10 D-glucose, adjusted to pH = 7.4 using NaOH. Coverslips were loaded with 25 nM mitoTracker Red (Thermo Fisher) + 25–50 nM Fluo-4-AM (Thermo Fisher) for 10 min, washed twice for 5 min with HEPES buffer. Per neuron, a single transport recording was performed, and only one neuron was measured per coverslip. To ensure representative sampling and reduce culture-specific variability, measurements were performed in coverslips from multiple independent cultures. For fast time-lapse imaging of action potential firing, neurons were loaded with 625 nM BeRST1 (Huang et al., 2015) (gift from Dr. Evan Miller) and 50 nM Fluo4. Traces were recorded by rapidly scanning (~300 Hz) a 2-by-4 grid on the edge between the cytosol and cell membrane to detect the intracellular calcium response (Fluo-4) and membrane voltage (BeRST1), respectively. To record mitochondrial transport in “connecting” axonal segments, 10 min recordings (0.2 Hz) in sparse mixed mito-dendra2:wildtype (50:50) cultures, with manually triggered stimulation after 1 min. Due to axonal branching, data from multiple segments of the axon of interest within the FOV were pooled for each recording (as the data could not be extracted in a single continuous line). The connection, as well as axon identity were confirmed using immunolabeling after each recording.

### Stimulation of neurons

At a set time during the recording, electrical field stimulation was applied via a miniature, custom-built bipolar electrode carefully positioned over the field of view, connected to a high current stimulus isolator (WPI, A385) and Master 8 (A.P.I), to generate the desired current amplitude (7.5 mA) and stimulus paradigm, respectively. Three stimulation types were used: 20 Hz 2 s (40 APs), 100 Hz 2 s (200 APs), 2 Hz 2 min (240 APs).

### Ratiometric calcium imaging

Fura-2 imaging of neurons was performed using a 20× objective lens on a Nikon Eclipse TE300 inverted widefield microscope equipped with a Polychrome V monochromator (TILL Photonics) and Sensicam-QE (PCO Imaging). Fura-2 was excited at 340 and 380 nm and images were captured using a custom-designed FURA filter cube (specs: central wavelength/width: EX: 405/150; DM: 495; EM: 525/50) to split excitation from emission. Time-lapse recordings were acquired at 4 Hz for 30 s to measure responses to 20 Hz (2 s) and 100 Hz (2 s) stimulations, and at 1 Hz for 210 s for the 2-min long 2 Hz stimulation, using TillVision software. Sparse hippocampal cultures were loaded with 0.5 μM Fura-2-AM for 20 min at 37°C in plate media, then washed 3× with 37°C HEPES. After a 5 min rest, 3 FOVs were measured per coverslip (stimulation types in random order) within a 20 min window under continuous perfusion of HEPES buffer at 37°C. After each experiment, MAP2 & TUBB3 labeling was used to distinguish dendritic and axonal compartments. To analyze these recordings, a custom pipeline was created, as described in Figure 3. For the intracellular measurement of maximal (*R*_max_) and minimal (*R*_min_) ratios, HEPES buffers containing 5 μM ionomycin +2 mM Ca^2+^, and 5 μM ionomycin +0 mM Ca^2+^ + 2 mM EGTA were used, respectively, (*K*_D_ Fura-2 = 228 nM). Custom MATLAB scripts were used to perform the ROI-and pixel-based quantifications of absolute calcium traces.

### Transport analysis

A custom Igor-toolbox (Wavemetrics) was used to generate kymographs and trace mitochondrial trajectories, according to a previously described method (Vanden Berghe et al., 2004). In-house MATLAB scripts were used to analyze transport parameters.

### Statistical tests

Prism (GraphPad) was used for statistical analysis: *p* < 0.05 (*), *p* < 0.01 (**), *p* < 0.001 (***). Bar graphs represent mean values with STD error bars. To compare two groups, in case of a normal distribution, unpaired *t* tests were conducted as a two-sided test, otherwise the Mann–Whitney *U* tests were performed. Plots showing binned parameters traces over time represent mean values with SEM error bars.

## Data Availability

The raw data supporting the conclusions of this article will be made available by the authors, without undue reservation.
