# Supplementary material for: Neuronal activity inhibits mitochondrial transport only in synaptically connected segments of the axon
Source: Front Cell Neurosci. 2024 Dec 4;18:1509283. doi: 10.3389/fncel.2024.1509283 (PMC11652138; doi:10.3389/fncel.2024.1509283)
Supplement: Supplementary file 1 [file Data_Sheet_1.docx]

**Supplementary Figure 1. Additional transport quantifications related to figure 1.** (a) Average anterograde vs. retrograde distance traveled during baseline period (1^st^ half of recording) vs. post-stimulation (2^nd^ half of recording). Same dataset as shown in figure 1g. Mann-Whitney U test, p = 0.92 (ctr antero), 0.93 (ctr retro), 0.98 (100 Hz antero), 0.61 (100 Hz retro), 0.81 (20 Hz antero), 0.51 (20 Hz retro). Quantification of mobile percentage before and after stimulation. Mann-Whitney U test, p = 0.52 (ctr), 0.76 (100 Hz), 0.91 (20 Hz). (c) Quantification of average instantaneous velocity (stationary periods excluded). Mann-Whitney U test, p = 0.99 (ctr), 0.53 (100 Hz), 0.56 (20 Hz). N = 30 recordings & 255 mobile mitochondria (ctr), 20 recordings & 145 mobile mitochondria (100 Hz), 23 recordings & 205 mobile mitochondria (20 Hz).
